# Neo-antigens for the serological diagnosis of IgE-mediated drug allergic reactions to antibiotics cephalosporin, carbapenem and monobactam

**DOI:** 10.1038/s41598-020-73109-w

**Published:** 2020-09-29

**Authors:** Edurne Peña-Mendizabal, Sergi Morais, Ángel Maquieira

**Affiliations:** 1 Instituto Interuniversitario de Investigación de Reconocimiento Molecular y Desarrollo Tecnológico, Universitat Politècnica de València-Universitat de València, Camino de vera s/n, 46022 Valencia, Spain; 2 Departamento de Química, Universitat Politècnica de València, Camino de Vera s/n, 46022 Valencia, Spain; 3 Unidad Mixta UPV-La Fe, Nanomedicine and Sensors, IIS La Fe, Valencia, Spain

**Keywords:** Analytical chemistry, Immunological disorders

## Abstract

New antigens deriving from -lloyl and -llanyl, major and minor determinants, respectively, were produced for β-lactam antibiotics cefuroxime, cefotaxime, ceftriaxone, meropenem and aztreonam. Twenty β-lactam antigens were produced using human serum albumin and histone H1 as carrier proteins. Antigens were tested by multiplex in vitro immunoassays and evaluated based on the detection of specific IgG and IgE in the serum samples. Both major and minor determinants were appropriate antigens for detecting specific anti-β-lactam IgG in immunised rabbit sera. In a cohort of 37 allergic patients, we observed that only the minor determinants (-llanyl antigens) were suitable for determining specific anti-β-lactam IgE antibodies with high sensitivity (< 0.01 IU/mL; 24 ng/L) and specificity (100%). These findings reveal that not only the haptenisation of β-lactam antibiotics renders improved molecular recognition events when the 4-member β-lactam ring remains unmodified, but also may contribute to develop promising minor antigens suitable for detecting specific IgE-mediated allergic reactions. This will facilitate the development of sensitive and selective multiplexed in vitro tests for drug-allergy diagnoses to antibiotics cephalosporin, carbapenem and monobactam.

## Introduction

Between 2000 and 2015, antibiotic consumption, expressed in defined daily doses (DDD), increased 65% (21.1–34.8 billion DDDs) and its rate rose 39% (11.3–15.7 DDDs per 1000 inhabitants/day). This increase was driven by low- and middle-income countries, where rising consumption has been correlated with gross domestic product per capita growth^1^. In fact β-lactams (BLCs) are the most widely used antibiotics for treating bacterial infections and, consequently, the most frequent cause of allergic reactions^2–5^. They represent one of the world’s major biotechnology markets with annual sales of around $15 billion, and make up around 65% of the total antibiotics market (world’s antibiotic sales of 3 × 10^7^ kg/year of a total 5 × 10^7^ kg/year produced worldwide)^6^. For many years, the most commonly prescribed and studied BLCs were penicillins^4,7,8^ like benzylpenicillin (PG), amoxicillin (AMX) or ampicillin (AMP). Due to the increased allergies related to penicillins, antibiotic research rapidly led to the discovery and use of several other BLC families^9^. Indeed the global antibiotic consumption rate of cephalosporins, carbapenems and monobactams were 135.45, 11.26 and 6.23 DDDs, respectively, between 2016 and 2018^10^. In fact the consumption of all these other BLCs in 2018 was 2.0 DDDs per 1000 inhabitants per day, which accounts for around 11% of the total European intake^11^.

The chemical structure differences among BLC antibiotics promote the allergenic recognition of a broad spectrum of immune system specificities and may therefore help to provoke adverse reactions. BLCs generate -lloyl determinants after β-lactam ring opening under physiologic conditions. According to the bibliography, this -lloyl determinant is considered to be the ‘major’ determinant for penicillins because it is responsible for almost 95% of all allergic reactions to penicillins^12^. The other determinants are classified as ‘minor’ or -llanyl determinants, and are associated with causing anaphylaxis in immediate immunoglobulin E (IgE)-mediated reactions in 95% of cases^13,14^, but the explanation for the direct involvement of these determinants in provoking immediate hypersensitivity reactions still remains unclear^15^. To date, many studies have focused on major determinants^16^. However, BLC allergic reactions caused by minor determinants have been considered extremely important in penicillin allergies; e.g., in skin tests for diagnosing β-lactam allergy^17^, and have been associated with specific systemic anaphylaxis^18^. For this reason, it is necessary to systematically and clinically study the relevance and relative importance of these -llanyl derivatives as a new range of ‘minor’ determinants for β-lactam allergy^13^. Several prospective studies are found in the literature that have evaluated the use of cephalosporins^4,5,19,20^, carbapenems^21^ or monobactams in patients with documented penicillin allergy who specifically require BLC treatment, but very few in vitro assays are available for these studies^3^. In any case, many patients supposedly allergic to a BLC, or at least present allergic episode to them, are usually classified as allergic to a drug with no further investigation^22^ or are not treated with BLCs because patients might be affected by them and at risk for anaphylaxis. The use of other antibiotics may contribute to the development of multiple drug-resistant bacteria, the emergence of other potentially dangerous side effects and a lead-in of higher healthcare costs^2^. Whatever the case, those patients self-reporting a penicillin allergy can be safely tested for the presence of a true allergy, normally by allergy diagnosis tests.

Allergy diagnoses must include a detailed clinical history, physical examination and in vivo tests (skin and/or drug provocation tests). However, these tests pose life-threatening risks and can entail false-positive skin test results^23^. Currently, the gold standard in vitro test is based on ImmunoCap technology, but it is available only for five BLCs: PG, phenoxymethyl penicillin, AMP, AMX, cefaclor. Therefore, good antigenic determinants are lacking to develop accurate immunoassays with the potential for determining specific IgE to other BLCs in order to cover other subfamilies of BLCs to improve clinical diagnoses. A good diagnosis that relies on both in vivo and in vitro tests could allow a significant delabelling of the reported BLC allergy, and the majority of patients tolerate BLCs without incidents^24^. Furthermore, effective immunoassays for allergy diagnosis rely on the selection of appropriate haptens and metabolites to elicit an immune response. An examination of the specificities of BLC-reactive IgE antibodies in sera revealed a heterogeneous group of allergenic determinants, probably because the authors did not take into account the fine structural heterogeneity of the allergenic determinants of the complete range of BLCs^25^. When designing determinants, it is important to retain the functional groups that are unique to hapten^26^.

In this work, the main objective was to design and produce major and minor -lloyl- and -llanyl-derived antigens, respectively, for cefuroxime (CFR), cefotaxime (CFO), ceftriaxone (CFT), meropenem (MRP) and aztreonam (AZT). Compounds were evaluated by a multiplex direct microimmunoassay developed on the Digital Versatile Disk (DVD) using sera from immunised rabbits, and allergic patients and controls. To the best of our knowledge, this is the first report of major and minor -lloyl and -llanyl antigens for these antibiotics, and this work demonstrates the potential for an improved serological diagnosis of IgE-mediated drug allergic reactions for commonly prescribed and consumed β-lactam antibiotics.

## Experimental section

### Chemicals, immunoreagents and buffers

**Figure 1:**
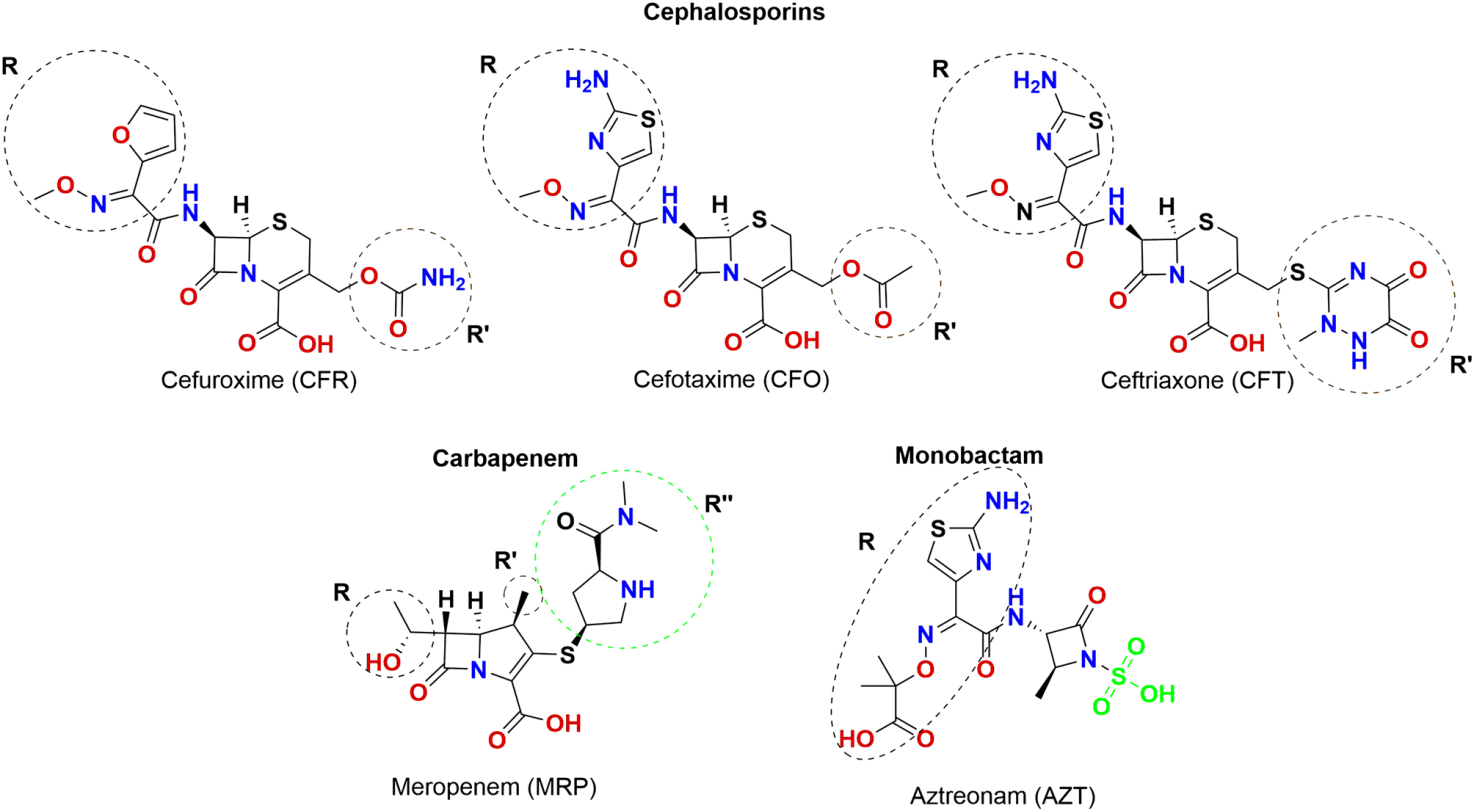
Chemical structures of the studied β-lactam antibiotics. ChemDraw Professional, Version 17.0.0.206 (121) https://www.perkinelmer.com/es/product/chemdraw-professional-chemdrawpro.

Benzylpenicillin sodium salt, meropenem trihydrate, aztreonam, cefuroxime sodium salt, cefotaxime sodium salt, ceftriaxone disodium salt hemi(heptahydrate), *N*,*N*′-dicyclohexylcarbodiimide (DCC), *N*-hydroxysuccinimide (NHS), Tween 20, human serum albumin (HSA), histone from calf thymus (H1) and keyhole limpet hemocyanin (KLH) came from Sigma-Aldrich (Madrid, Spain). The chemical structures of the studied BLCs are shown in Fig. 1. Dichloromethane (DCM), dimethylformamide (DMF), hydrochloric acid 37% (HCl) and buffer salts were purchased from Scharlau (Sentmenat, Spain) and used without further purification. Deuterated dimethyl sulfoxide (DMSO-d_6_) was supplied by ACROS Organics (New Jersey, USA). The anti-human IgE monoclonal antibody was purchased from Ingenasa, S.A. (Madrid, Spain). The goat anti-rabbit antibody labelled with horseradish peroxidase (GAR-HRP) and the goat anti-mouse antibody labelled with horseradish peroxidase (GAM-HRP) were supplied by Abcam (Cambridge, UK). IgE human serum (3rd WHO International Standard) was bought from the National Institute for Biological Standards and Control (NIBSC, Hertfordshire, UK). The tetramethylbenzidine (TMB) substrate came from SDT GmbH (Baesweiler, Germany) and Coomassie Brilliant Blue R-250 staining solution was acquired from Bio-Rad (Madrid, Spain). Amicon Ultra 0.5 pre-concentred 10K MWCO filters and Dextran Desalting Columns, 5K MWCO, 5 mL were from Fisher Scientific (Madrid, Spain).

Buffers were: (I) potassium carbonate 0.5 M, pH 11.0; (II) phosphate buffer saline (PBS 1 ×, 0.008 M sodium phosphate dibasic, 0.002 M sodium phosphate monobasic, 0.137 M sodium chloride, 0.003 M potassium chloride, pH 7.4); (III) PBS-T (PBS 1 × containing 0.05% Tween 20); (IV) sodium carbonate/bicarbonate buffer 0.1 M, pH 9.6, as the printing buffer. All the buffers were filtered through a 0.45-μm pore size nitrocellulose membrane from Thermo Fisher Scientific (Madrid, Spain) before being used.

### Acidification of cephalosporin salts

Firstly, cephalosporin salts were acidified as follows: a round-bottomed flask was filled with a solution of the corresponding BLC salt dissolved in water. Then the solution was acidified with HCl 6 M followed by filtration in a vacuum. The compound was washed twice with acidified water and dried in a high vacuum to obtain the desired acid. The detailed procedures and data characterisation of all the acidified cephalosporins are shown in the online Supplementary Information (SI).

### Preparation of the structural antigens

**Figure 2:**
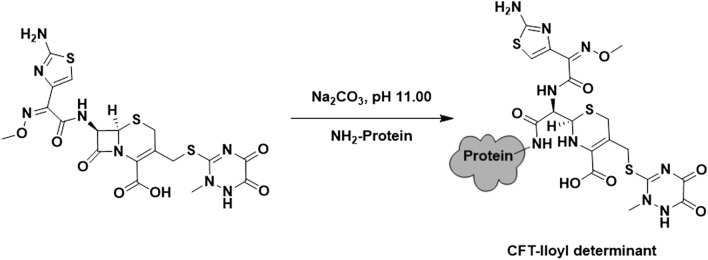
Conjugation of protein CFT -lloyl determinant. https://www.microsoft.com/es-es/microsoft-365/powerpoint.

KLH was used to produce the immunogenic antigens (-lloyl and -llanyl), with which New Zealand white rabbits were immunised to raise specific IgG for CFT as a model of cephalosporin, MRP and AZT. Two carrier proteins, HSA and H1, were employed to produce the antigens. The preparation was as follows:

The β-lactam-lloyl determinants were linked through the lysine residues of the carrier proteins by β-lactam ring opening, through the amidation between the carbonyl carbon of the β-lactam ring and the amino group of the lysine residues, as previously described^27^ with a few modifications (Fig. 2). In short, the carrier protein (2.0 mg) dissolved in potassium carbonate 0.5 M, pH 11.0, was reacted with the corresponding BLC salt (22–30 μmol depending on the used protein) overnight at room temperature.

**Figure 3:**
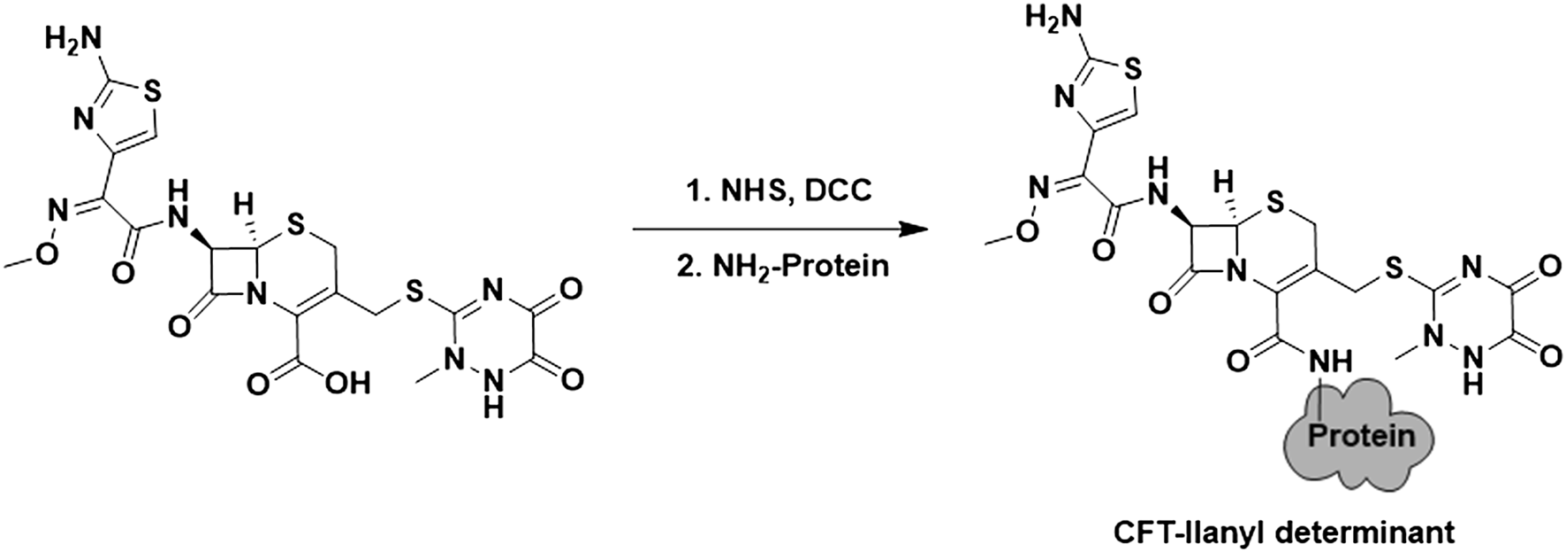
Conjugation of protein CFT -llanyl determinant.

The conjugation of the β-lactam -llanyl determinants was performed following the carbodiimide chemistry^28^ (Fig. 3). For that purpose, 22–30 μmol (depending on the employed carrier protein) of the corresponding BLC as its free acid were reacted with 55 μmol of NHS and DCC in DMF (500 μL) for 4 h at room temperature. Afterwards, the mixture was centrifuged at 12,000 rpm to remove the acyl urea precipitate. Finally, 250 μL of the supernatant were added to 2.25 mL of the carrier protein solution (2.0 mg) in PBS 1 ×, pH 7.4, for 4 h at room temperature.

Instead of the chemical structure of AZT having a free carboxylic acid, it had a sulfonate moiety attached to the β-lactam ring (Fig. 1). For this reason, the AZT-llanyl determinant was prepared with the free carboxylic acid presented in its side chain following the same procedure as described for the -llanyl determinants.

The HSA and H1 -lloyl and -llanyl determinants were purified by gel filtration chromatography using Amicon Ultra 0.5 pre-concentred 10 K filters and PBS 1X, pH 7.4, as the elution buffer. The KLH -lloyl and -llanyl determinants were purified by size exclusion chromatography on dextran desalting columns using PBS 1 ×, pH 7.4, as an elution buffer.

All the determinants were diluted to 1.0 mg/mL and stored at – 20 °C until used. The concentration of the determinants was established by the Bradford protein assay^29^. The β-lactam/carrier protein molar ratio for the HSA determinants was established by MS-MALDI-TOF^30^. Histone H1 is an isolated lysine-rich fraction of mainly subfraction f1, with the other subfractions still present. The H1 antigens could not be analysed by MS-MALDI-TOF. However, antigens HSA and H1 were prepared following the same experimental procedure and according to the formation of the major or minor determinants. As both proteins presented approximately 60 free lysine residues, the molar ratios of antigens H1 were estimated to be the same as those obtained for the respective HSA antigens. The KLH determinants were difficult to characterise because of the protein’s high molecular weight. The selectivity of the raised rabbit IgGs was tested during the immunisation by the dot blot technique^31^ and the conjugation was considered positive when the sera obtained from the immunised rabbit specifically recognised the corresponding antigen.

### Assay protocol to evaluate antigens

**Figure 4:**
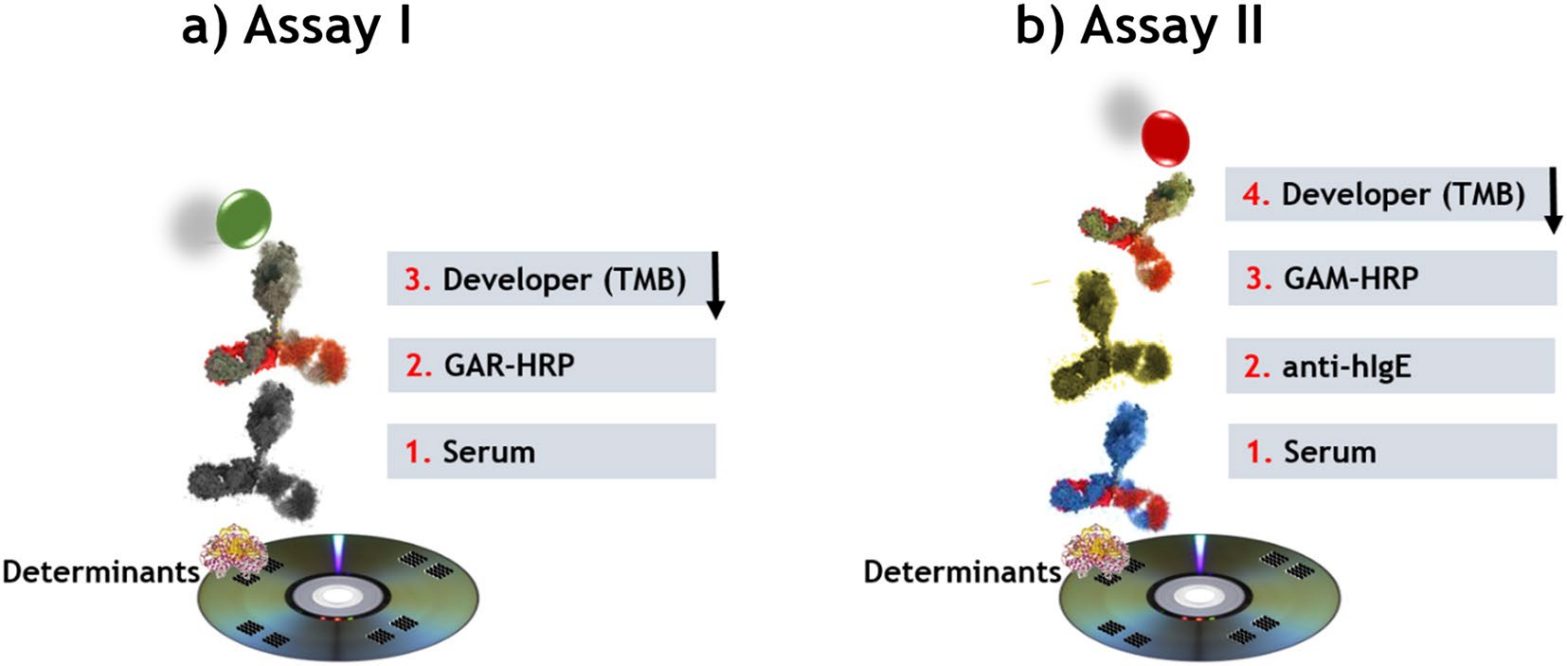
Scheme of the multiplexed microimmunoassays I and II based on a direct format with colorimetric detection.

**Figure 5:**
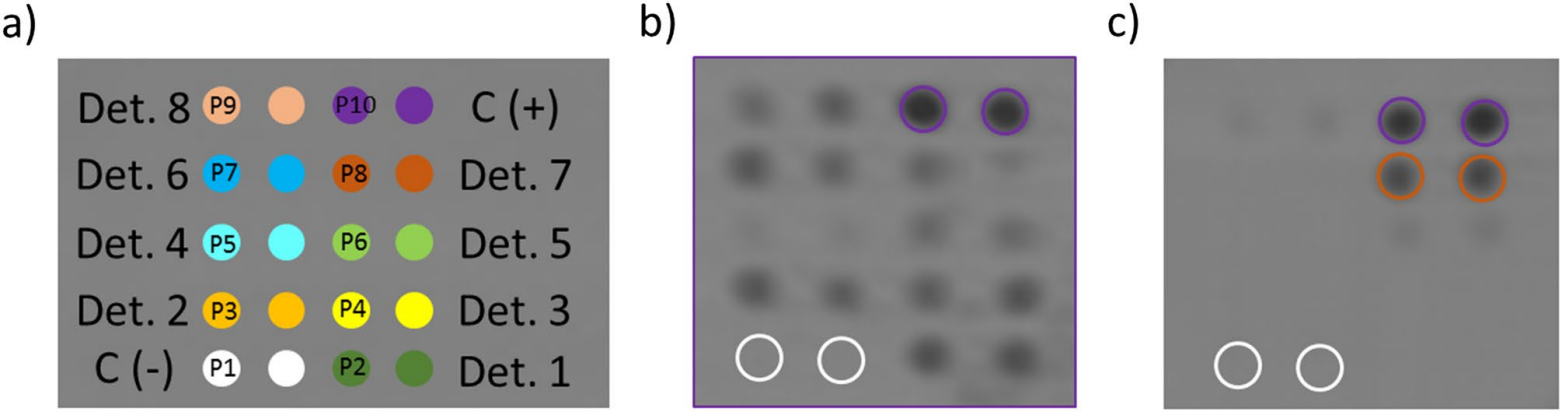
(**a**) Layout of the multiplexed microarray. Position 1: HSA, negative control, C(−); and Position 10: rIgG or hIgE, positive control, C(+); (**b**) Image of an array on the DVD after immunoassay I with α-IgG to CFT, dilution factor 1/1,000 (Position P1: HSA, negative control; P2: CFT-HSA-llanyl determinant; P3: CFT-HSA-lloyl determinant; P4: CFT-H1-lloyl determinant; P5: CFR-HSA-llanyl determinant; P6: CFO-H1-llanyl determinant; P7: CFR-H1-lloyl determinant; P8: CFR-H1-llanyl determinant; P9: CFO-H1-llanyl determinant; P10: rIgG); (**c**) Representative result of the array with specific determinants and controls printed on the DVD after immunoassay II with patient 002 (determinants in the same positions as in (**b**), except for P10: hIgE).

The assays consisted in detecting specific IgG (Fig. 4a, assay I) and IgE (Fig. 4b, assay II) on standard DVDs (CD Rohling-up GmbH, Saarbrücken, Germany). To this end, determinants (40 µg/mL) and controls (negative and positive), prepared in printing buffer, were spotted in a microarray format (20 arrays per disk of 5 × 4 spots) by dispensing 25 nL of each one using a non-contact printing device (AD 1500 BioDot, Inc., Irvine, CA, USA). Spots were 500 μm in diameter with a centre-to-centre distance of 1.0 mm. In each microarray (Fig. 5), the spots for the produced determinants (two replicates, position 2–9) and the negative (HSA, position 1) and positive (rabbit IgG or human IgE, position 10) controls were included. After printing, the DVD was incubated for 16 h at 37 °C.

To detect specific IgG against CFT, MRP and AZT, different dilutions (1/250–1/16,000) of the rabbit sera and control (PBS-T) (25 µL per sample) were added to each array and incubated for 15 min. Then the DVD was washed with PBS-T and water before adding 25 µL of polyclonal secondary antibody GAR-HRP in PBS-T buffer (dilution 1/400) for 15 min, followed by the washing step. To detect specific IgE, 25 µL of the sample (allergic patients and controls) was added to each array and incubated for 30 min. After washing, 25 µL of the mAb-IgE in PBS-T buffer (1 µg/mL) were added and incubated for 15 min. After washing like before, 25 µL of a 1/100 dilution of GAM-HRP were added for 15 min. Finally, an immunoreaction was run in both immunoassays by homogeneously dispensing 1.0 mL of TMB over the entire disc surface. The reaction was stopped by washing the disk with water after 15 min. Signals were read by a modified DVD drive and data were analysed as previously described^32,33^. All the experiments were repeated 3 times.

The affinity of the specific IgGs to CFT, MRP and AZT towardsthe antigens was calculated by measuring the apparent affinity constant (K_d_^app^) in the saturation assays. K_d_^app^ represents the apparent equilibrium dissociation constant between specific IgG and the corresponding antigenic determinant, and is related to the in vitro concentration (expressed as the dilution factor) of the specific IgG antibodies that reached half the maximum signal in the saturation assay. Binding curves were fitted by the SigmaPlot 11 software.

The BLC-specific IgE levels expressed as units of specific IgE (IU/mL) were determined by the 3rd WHO standard for total serum IgE determinations, which involves heterologous interpolation as a calibration scheme. The calibration curve was built by performing a sandwich immunoassay where the 3rd WHO International Standard was used as a calibrator and Omalizumab^34^ as the capture antibody. All the other immunoreagents were the same as those used for determining specific IgE (assay II). The standard data points, signal versus semi-log concentration, were the mean of 10 curves performed on different disks on distinct days. A four-parameter logistic (4PL) curve was fitted (Supp. Fig. S1) using the SigmaPlot 11 software. The specific IgE concentrations were calculated from the calibration curve for the total IgE.

### Serum samples

The study of the reactivity of the prepared determinants to the sera from the allergic patients included those patients (I) who had been diagnosed with an immediate allergic reaction to a BLC by prick test;s (II) whose culprit drug was one BLC (e.g. antibiotics amoxicillin, augmentin, benzylpenicillin, cefuroxime and ceftriaxone); (III) with the total IgE (tIgE) values between 100 and 400 IU/mL (clinically considered the normal range for total IgE in a healthy population^35^). The clinical characteristics of the 37 patients included in this study are shown in Table 1. Thirty-seven subjects with negative skin tests to BLCs and good tolerance to them were used as controls. Their clinical characteristics are shown in Table S1. All the serum samples were kindly provided by the Hospital Universitari i Politènic La Fe in Valencia, Spain. All participants were enrolled after giving written informed consent according to protocols approved by the ethics review board at La Fe University Hospital (registry no. COBIOPHAD). The procedures followed were in accordance with the Helsinki Declaration of 1975 as revised in 2008. The patients were diagnosed following the procedure described in the European Network of Drug Allergy (ENDA) protocol based on skin testing, in vitro tests or drug provocation test, whenever necessary.

**Table 1:** Clinical characteristics of the cohort of allergic patients.

Patient number	Gender	Age (years)	Culprit Drug	Clinical manifestation	Route	Timing
001	M	68	Augmentin	Anaphylaxis	Parenteral	Immediate
002	F	46	Cefuroxime	Anaphylaxis	Oral	Immediate
003	F	53	Cefuroxime	Cutaneous	Oral	Delayed
004	M	49	Augmentin	Cutaneous	Oral	Delayed
005	M	80	Augmentin	Anaphylaxis	Parenteral	Immediate
006	F	37	Cefuroxime	Cutaneous	Oral	Immediate
007	M	40	Augmentin	Cutaneous	Oral	Immediate
008	F	24	Amoxicillin	Cutaneous	Oral	Immediate
009	M	27	Ceftriaxone	Cutaneous	Parenteral	Immediate
010	F	68	Augmentin	Anaphylaxis	Parenteral	Immediate
011	F	49	Augmentin	Anaphylaxis	NR	Immediate
012	F	52	Augmentin	Anaphylaxis	Oral	Immediate
013	M	61	Augmentin	Cutaneous	Oral	Delayed
014	F	34	Cefuroxime	Anaphylaxis	Oral	Immediate
015	M	54	Amoxicillin	Anaphylaxis	Oral	Immediate
016	M	10	Amoxicillin	Unspecific	Oral	NR
017	F	41	Amoxicillin	Cutaneous	Oral	Immediate
018	M	45	Augmentin	Cutaneous	Oral	Delayed
019	F	53	Augmentin	Cutaneous	Oral	Immediate
020	M	39	Amoxicillin	Cutaneous	Oral	NR
021	F	56	Augmentin	Cutaneous	Oral	Immediate
022	F	68	Amoxicillin	Unspecific	Oral	Immediate
023	M	40	Penicillin G	Cutaneous	NR	Delayed
024	M	53	Augmentin	Anaphylaxis	Oral	Immediate
025	F	49	Ceftriaxone	Anaphylaxis	Parenteral	Immediate
026	F	53	Augmentin	Cutaneous	Oral	Immediate
027	M	35	Amoxicillin	Anaphylaxis	Oral	Immediate
028	F	36	Augmentin	Cutaneous	Oral	Immediate
029	F	54	Augmentin	Cutaneous	Oral	NR
030	F	46	Augmentin	Anaphylaxis	Oral	Immediate
031	F	71	Augmentin	Cutaneous	Oral	Immediate
032	F	32	Cefuroxime	Anaphylaxis	Oral	NR
033	F	55	Cefuroxime	Cutaneous	Oral	Delayed
034	F	39	Cefuroxime	Cutaneous	Oral	Delayed
035	M	46	Augmentin	Anaphylaxis	Oral	Immediate
036	F	80	Cefuroxime	Anaphylaxis	NR	Immediate
037	F	72	Cefuroxime	Cutaneous	NR	Immediate

The terms immediate and delayed refer to an allergic reaction provoked by re-exposure to a specific allergen and to late-phase allergic reactions that generally occur between 2 and 6 h after exposure to a specific allergen, respectively.

*F* female, *M* male, *NR* not reported in the clinical history.

## Results and discussion

### Production of β-lactam antigens

Firstly, acidification of cephalosporin salt (CFR, CFO and CFT) was attempted to obtain the corresponding carboxylic acid. The reaction proceeded easily with a high yield (ca. 90%). The purity of the acidified BLC was high (see the NMR spectra in the Supplementary Information). The production of antigens relied on the conjugation of the acidified BLC (CFR, CFO, CFT, MRP and AZT) to the carrier proteins^36,37^. In this study, human serum albumin and histone H1 were used, and both presented approximately 60 free lysine residues to approach two different conjugation routes in order to obtain the corresponding major and minor determinants for each β-lactam antibiotic. The initial molar ratio (β-lactam/carrier protein) was set at 1000/1 and 240/1 for HSA and H1, respectively, to ensure the conjugation of antibiotics. No elucidation method was followed for the characterisation of the determinants. However, according to the MS-MALDI-TOF results (see the online Supplementary MS-MALDI-TOF spectra), the molar ratio of the HSA antigens ranged from 1 to 8. In particular, the molar ratios of both the aztreonam and ceftriaxone major and minor antigens were similar, whereas the molar ratios were 1 and 8, respectively, for the meropenem antigens. In general, the conjugation yield was very low for the HSA antigens (< 0.8%). This could be due to the protecting-group-free production of the -llanyl antigens. Most of the studied BLCs bear a primary amine group in their R substituent, which could compete with that amino from Lys to form some kind of oligomers of β-lactam by amidation.

### Evaluation of antigens

The antigens were firstly evaluated as a proof of concept using sera raised to both the -loyl and -llanyl antigens for CFT, MRP and AZT. As observed in Supplementary Figs. S2–S4, the sera obtained from the immunised rabbit with the -lloyl-based determinants specifically recognised the corresponding antigen in the dot-blot assay. Conversely, this was not the case with the -llanyl determinants, which showed no specific recognition. For instance, as seen in online Supplementary Fig. S4, the AZT sera obtained from the immunised rabbit with the -lloyl-based determinant specifically recognised both the -lloyl and -llanyl determinant antigens at a dilution factor of 1/100 (v/v), but only recognised the AZT -lloyl antigen when the dilution factor was 1/500 (v/v). However, the AZT sera obtained with the -llanyl determinant did not recognise any determinant at any of the studied sera dilutions.

**Figure 6:**
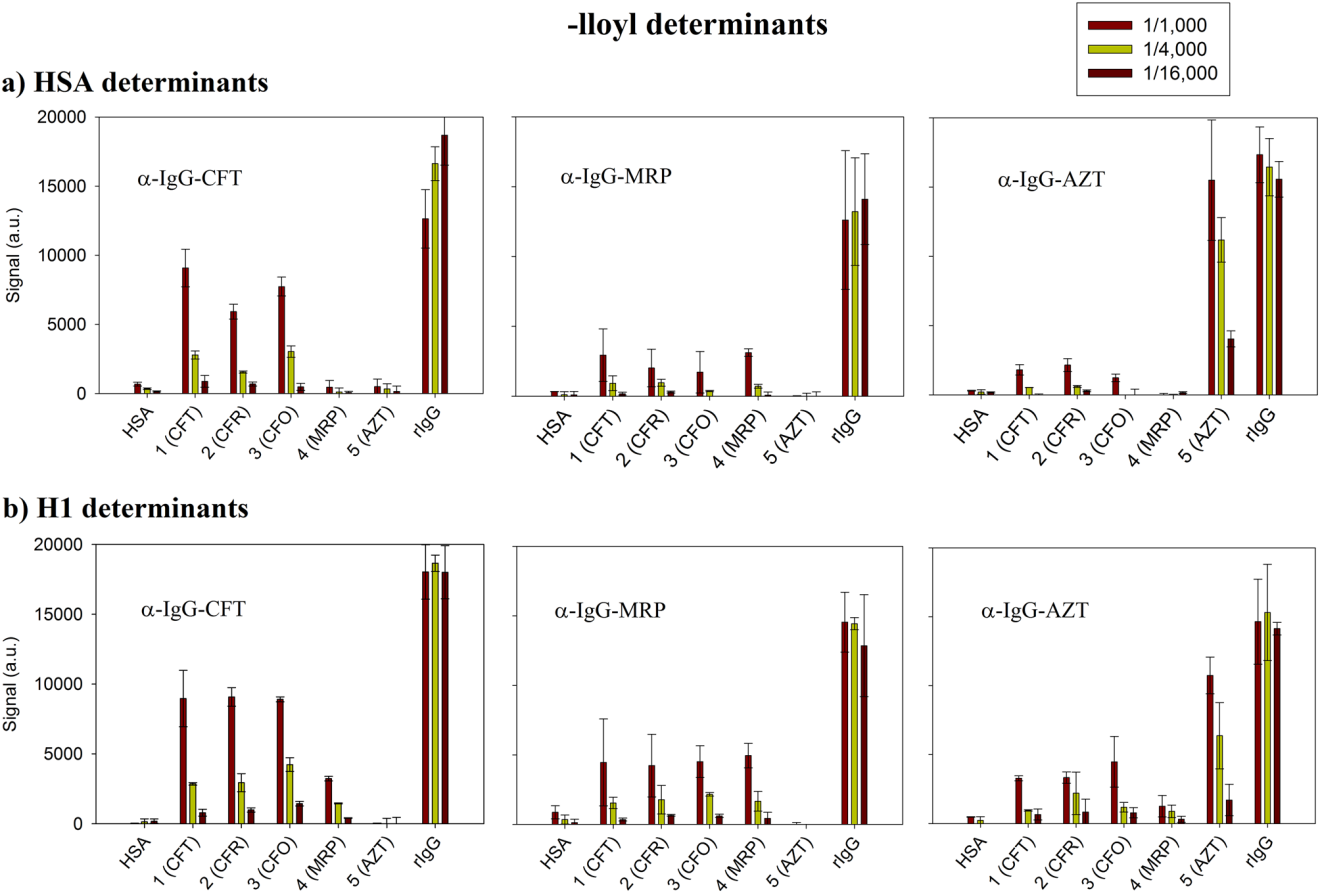
Signals obtained for the -lloyl determinants with polyclonal rabbit IgG. Dilution factors: 1/1000, 1/4000 and 1/16,000). (**a**) HSA determinants; (**b**) H1 determinants.

**Figure 7:**
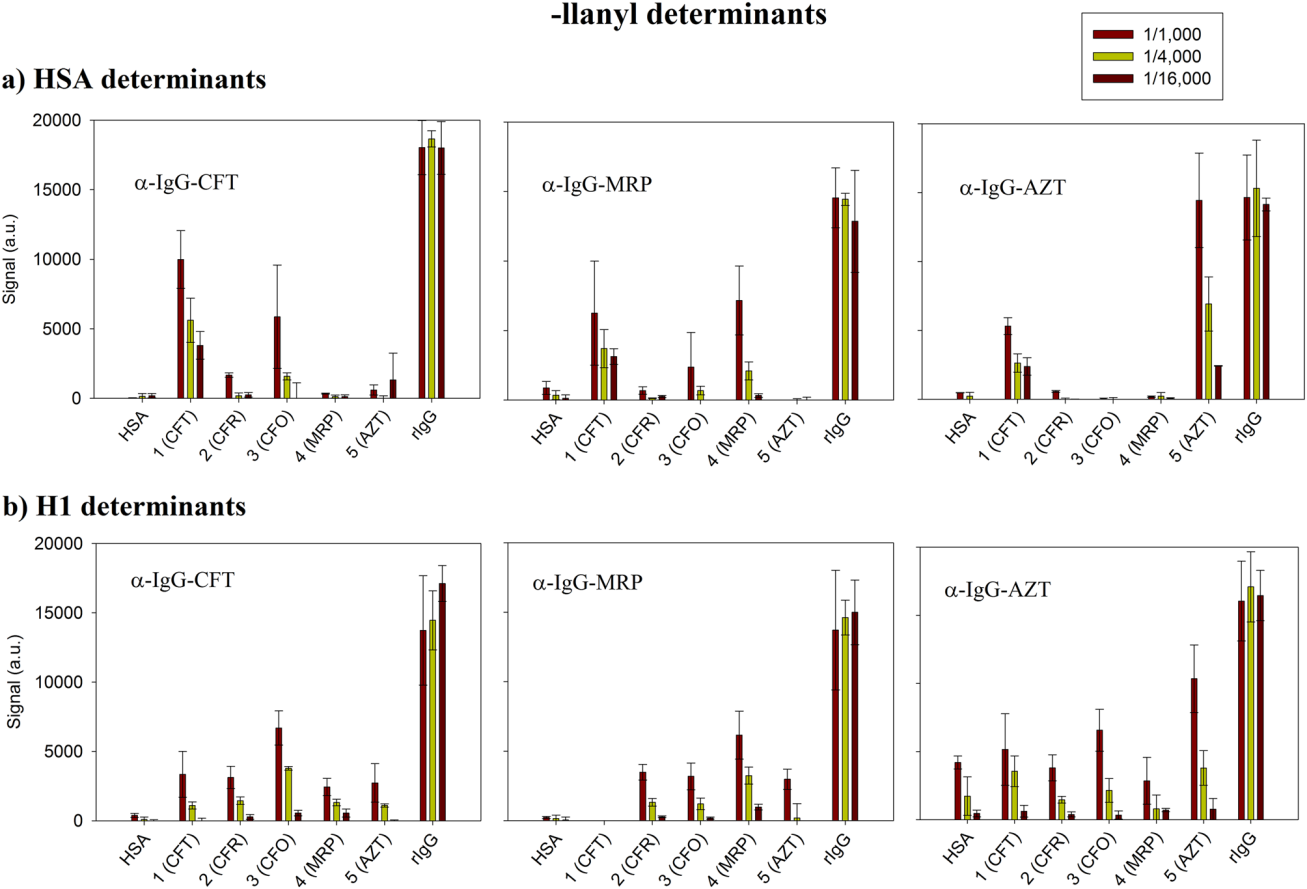
Signals obtained for the -llanyl determinants with polyclonal rabbit IgG. Dilution factors: 1/1000, 1/4000 and 1/16,000). (**a**) HSA determinants; (**b**) H1 determinants.

In order to evaluate the effect of the conjugation on assay sensitivity, each -loyl and -llanyl antigen was screened by a direct multiplex immunoassay with the -lloyl immunised sera to CFT, MRP and AZT. The tested sera dilutions were 1/250, 1/1000, 1/4000 and 1/16,000, while PBS-T was used as a blank. The obtained results are plotted in Figs. 6 and 7 for the -lloyl and -llanyl determinants, respectively. The values represented the mean of the signals (n = 3) obtained in each assay, together with the standard deviation (SD) expressed as error bars. As shown in the figures, the signals corresponding to the positive control (rIgG) were around 15,000 a.u. (± 8%) for all studied sera with no recognition observed for HSA (< 500 a.u) used as a negative. The dilution of the different sera allowed immunoassay sensitivity to be estimated. As we can see in Fig. 7b, using sera raised to MRP (α-IgG-MRP) detected the meropenem -llanyl antigen up to the 1/16,000 dilution, and gave a signal value of 1500 a.u, which corresponded to the limit of detection.

When focusing on the 1/1000 dilution, the response and cross-reactivity between the determinants for the different rabbit sera were compared. As we can see in Fig. 6, the -lloyl determinants for cephalosporin (CFR, CFO, CFT) were specifically identified mostly by the serum raised for CFT and no cross-reactivity was observed for AZT and MRP. A similar pattern was detected when using the AZT determinants and the serum raised for it. Indeed as expected, no other produced determinants were recognised, which indicates that recognition was specific for monobactams. Conversely, the MRP-derived determinants were recognised mostly by the corresponding serum, but values were similar to those obtained for the cephalosporin antigens, which suggests than the selectivity of the serum raised in rabbit was not high.

As far as cross-reactivity was concerned, there was evidence for different patterns with the case -llanyl determinants (Fig. 7), depending on the used carrier protein. As we can see, the serum raised for CFT mostly detected the antigens produced for CFO and no cross-reactivity to AZT and MRP was identified. However, the results were the opposite for CFR due to the poor recognition of these determinants in any studied immunisation sera. With CFT, the serum raised for it actually detected the HSA-antigen and cross-reactivity against other sera. In general, the sera increased for MRP and AZT detected the -llanyl MRP and AZT antigens, respectively, with no cross-reactivity to other BLCs, although AZT responses were higher than those observed with MRP.

In order to study the affinity of the structural determinants to the serum raised for the different β-lactam families, the binding values were analysed. The results are provided in online Supplementary Figs. S5, S6. The affinity values (as K_d_^app^ and coefficient of determination, R^2^) for all the rabbit IgG antibodies to each antigenic determinant are shown in online Supplementary Tables S2, S3. The K_d_^app^ values ranged from 10^–4^ to 10^–2^ (dilution factors from ~ 1/1000 to ~ 1/20), whereas R^2^ ranged between 0.9605 and 0.9994 and 0.6111 and 0.9984 for the -lloyl and -llanyl determinants, respectively.

It can be concluded that the HSA -lloyl determinants for CFR and AZT showed the best affinity (higher dilution) to the sera raised against CFT and AZT with K_d_^app^ values corresponding to a dilution factor of 1/1011 and 1/5587, respectively. However, the H1-lloyl determinants for CFT and CFO showed the best recognition to the sera raised against ceftriaxone at dilution factors of 1/1157 and 1/2271, respectively.

For the HSA -llanyl determinants, AZT showed the best affinity to the sera raised against it with a K_d_^app^ value corresponding to a dilution factor of 1/2228. However, the H1 -llanyl CFT and MRP determinants indicated the best recognition to the sera raised against them with K_d_^app^ values corresponding to dilution factors of 1/2168 and 1/1437, respectively.

Even though some determinants had an affinity to serum, this did not necessarily mean that they gave a good response^38^; e.g., this is the case with H1 -llanyl antigens CFT and CFO to the sera raised against AZT. It was generally concluded that the prepared major and minor determinants were selective to the recognised specific IgGs, with significant differences between the employed carrier proteins.

### Evaluation of the structural determinants with human serum samples

A cohort of 74 subjects was studied, of whom 37 positively developed an immediate reaction to BLC. According to the clinical data available for the allergic patients, 14 were men and 23 were women aged 10 and 80 years. The most frequent culprit drug was augmentin, a combination of AMX and potassium clavulanate (12 subjects), followed by CFR (9), AMX (7), CFT (2) and PG in one subject (Table 1).

The results of the prick test and the multiplex immunoassay are described in Table 2. It is worth mentioning that all the -lloyl and HSA -llanyl determinants did not render any positive results, with values below the LOD (< 0.01 IU/mL). Only the values given by the H1 -llanyl determinants are included in Table 2.

**Table 2:**
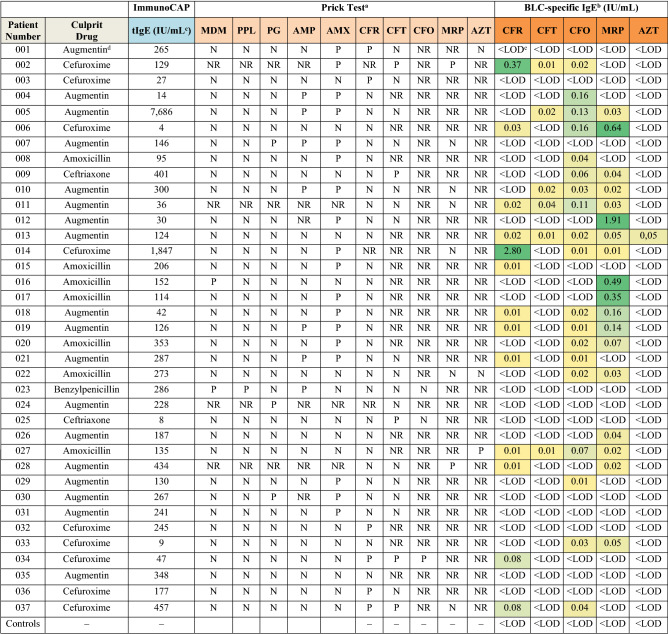
Results of analysing the human serum samples by in vivo (prick test) and in vitro (multiplex DVD assay) tests.

*MDM* minor determinants mixture (benzylpenicillin, sodium benzylpenicilloate and benzylpenicilloic acid), *PPL* penicillin major determinant (benzylpenicilloyl poly-l-lysine), *PG* benzylpenicillin, *AMP* ampicillin, *AMX* amoxicillin, *CFR* cefuroxime, *CFT* ceftriaxone, *CFO* cefotaxime, *MRP* meropenem, *AZT* aztreonam. Immunoassay values are the mean of three replicates. Relative standard deviation (RSD) ranged from 4 to 13%.

^a^*P* Positive, *N* Negative, *NR* Not reported in the clinical history.

^b^Multiplex-DVD assay using the H1–llanyl determinants.

^c^IU/mL = 2.4 ng/mL.

^d^Augmentin is a combination of amoxicillin and potassium clavulanate.

^e^< *LOD* Value below the limit of detection (LOD).

Firstly, it is important to highlight the lack of clinical information available for skin prick-tests for most of the studied antibiotics. Skin tests were performed only for CFR, and sometimes for CFT. In order to assess tests’ clinical sensitivity and selectivity, a gold standard is needed, defined as the diagnostic method that can discriminate patients with an allergic reaction to BLCs from those without one. It is difficult to calculate the sensitivity of skin tests because drug provocation tests cannot be performed as a gold standard to classify subjects as allergic or not allergic because of the high risk they entail^39^.

The skin test results showed that 6 of the 33 patients for CFR (18%), 3 of the 19 for CFT (29%) and 0 of the 2 for CFO were positive. As far as MRP and AZT were concerned, only 2 and 1 of 7 and 3 patients were skin-tested, which corresponded to 29% and 33%, respectively. It is noteworthy that the skin-tested patients to CFO, MRP and AZT obtained low percentage values (in terms of being positive to skin tests *versus* all the skin-tested patients), which must not be considered a general statement.

In order to determine the LOD and BLC-specific IgE levels, a sandwich immunoassay for total serum IgE determinations was performed as a calibration scheme. As the LOD of the multiplex immunoassay was 0.01 IU/mL (online Suppl. Fig. S2), which corresponded to 24 ng/L of specific IgE, we chose this LOD as the cut-off threshold for evaluation purposes. When the determinants with the sera of the 37 recruited patients were evaluated, 13 for CFR (35%), 6 for CFT (16%), 19 for CFO (51%), 18 for MRP (49%) and 1 for AZT (3%) were positive. The mean IgE values ± SD of the studied BLCs were 0.27 ± 0.77, 0.02 ± 0.01, 0.05 ± 0.05, 0.23 ± 0.46 and 0.05 ± 0 IU/mL to CFR, CFT, CFO, MRP and AZT, respectively. All the 37 control patients recruited in this study tested negative. Finally, no correlation was found between the total IgE levels from patients and the BLC-specific IgE levels.

Regarding the culprit drug that caused the allergic reaction, for CFR only five patients tested positive by prick tests, two were negative and two were not tested (see Table 2). The H1 CFR -llanyl determinant detected five patients diagnosed as positive for CFR with values ranging from 0.03 to 2.80 IU/mL. Interestingly, this antigen detected patient 006 that tested negative by prick tests. The IgE values for CFR were 0.37 and 2.80 IU/mL for patients 002 and 014, respectively. Fortunately, no cross-reactivity was observed for patient 014 to CFR, CFO, CFT, MRP or AZT. However, patient 002 was tested positive by skin tests for CFT and MRP. As the results of the skin tests and BLC-specific IgE quantifications differed, special attention was paid to these patients owing to the high health risk for CFR administration and the possibility of cross-reactivity. With patients 009 and 025, for whom CFT provoked an allergic reaction, our determinant was not selective enough to detect it, but both patients were skin-tested positive.

To CFO, MRP and AZT, none of the studied patients had an allergic episode. Of the 17 patient samples with specific IgE values to MRP, four had specific IgE values above 0.35 IU/mL (patients 006, 012, 016 and 017) and two between 0.10 and 0.20 IU/mL (patients 018 and 019). With CFO, of the 15 positive patients detected, four had specific IgE values between 0.10 and 0.20 IU/mL (patients 004, 005, 006 and 011). It is emphasised that none of these patients was skin-tested for MRP nor CFO. This fact could have potential effects because patients can be affected by them or be at-risk for anaphylaxis.

The purpose of our study was to produce a panel of major and minor determinants for CFR, CFO, CFT, MRP and AZT to develop a multiplex in vitro assay to detect specific IgE for these BLCs. We not only produced a panel of determinants, but also worked on developing a panel of possible epitopes using different carrier proteins. Firstly, our results with patients showed that no major (-lloyl) determinants were detected in any of the selected subjects, nor in the HSA-based determinants. These results reveal the importance of the conjugation route and the selection of the appropriate carrier protein. However, it cannot exclude HSA as a carrier protein candidate with immunogenic properties or -lloyl determinants as antigenic determinants to be used in further studies as this study followed a specific conjugation methodology. Besides, a good correlation was also found between positive in vitro immunoassay responses to CFR and skin test results, which reveals that the produced determinant is specific and can be used for in vitro diagnosis, especially if we consider that both assays (specific IgE quantification and prick test) confirmed five positive patients. Moreover, the multiplex assay with the appropriate antigens was able to detect specific IgE values to CFR in one patient with a negative prick-test result (patient 006) and in two patients in whom prick tests were not performed (patients 002 and 014). Therefore, IgE assays might be suitable when skin tests have not been done, when using non-soluble BLCs, with severe reactions or with high-risk patients. In fact, for MRP for which no skin tests were performed, four patients were positive according to the specific IgE amount determined with values above 0.35 IU/mL (patients 006, 012, 016 and 017) with the meropenem determinant. This confirmed the utility of the multiplexed assay for drug allergy diagnoses.

The use of in vivo and in vitro tests to diagnose allergic patients to BLCs needs to be adapted to the current scenario in which the consumption pattern of these antibiotics has changed, and penicillins are being progressively replaced mainly with other antibiotics like cephalosporins, carbapenems, monobactams, quinolones and tetracyclines^40^. Some authors suggest that skin testing with antigenic determinants of penicillins is no longer sufficient for evaluating patients for BLC allergy and that cross-reactivity between penicillins and cephalosporins^41^ or carbapenems^12^ can exist. For instance, cephalosporins with identical R side chains should be avoided for patients believed to be selectively allergic to aminopenicillins (e.g. cefatrizine and cefadroxil for amoxicillin or cefaclor for ampicillin)^42^. However, these patients may receive other cephalosporins that can also promote an allergic episode^43^, which could be avoided by analysing these sera by a multiplex in vitro assay.

In our study, specific IgE levels to CFT and MRP were observed in the patients whose culprit drug was augmentin or AMX. This was the case, for instance, of patients 012, 016 and 017 whose specific MRP IgE values ranged from 0.35 to 1.91 IU/mL. Cross-reactivity between CFR and MRP was noted in patient 006 whose culprit drug was CFR and was positive to MRP (IgE = 0.64 IU/mL). Data about related to cross-reactivity between cephalosporins and carbapenems is insufficient, but some authors suggest that carbapenems should be considered potentially cross-reactive with cephalosporins because of similarities to the β-lactam ring^44^. In patient 006, IgE antibodies were probably directed against the β-lactam ring, a common nuclear determinant shared by all BLCs, but no recognition was observed for CFT. The acetyl and carbamoyl groups of the R′ lateral chain of cefotaxime and cefuroxime, respectively, can be easily hydrolysed^45^ when producing antigens. However, the thiotriazinedione moiety of CFT is stable and may take part in the epitope of the antigen, which was probably the reason for the negative response to CFT.

Consequently, it is necessary to use a panel of BLCs because there are no clinically approved validated reagents. A well-established multiplex in vitro method was herein followed to evaluate the presence of IgE antibodies in a defined allergic population in which augmentin, CFR and AMX were the drugs that most often induced immediate reactions. As some authors suggest that anaphylaxis predominates the cutaneous condition^46^ (presented as urticaria) in allergic reactions to BLCs, using a multiplex in vitro test is a good alternative to in vivo assays to detect positive patients in short times and to study a conjunction of BLCs together and instantaneously given this multiplex capacity. The problem with false-negatives is also noteworthy. For instance, Demoly and Romano indicate a positive provocation test for 8–17% of patients with negative skin tests to penicillin^22^. Therefore, in those patients who are shown negative by skin and/or drug provocation tests, the combination of both in vivo and in vitro tests is a useful approach to detect all the potential allergic patients.

## Conclusion

The production and evaluation of structural determinants for β-lactam allergy is key for developing sensitive and selective in vitro tests to determine specific IgE that can complement in vivo tests, data and clinical history, whenever possible. The conjunction of both techniques can allow better diagnoses and allow significant delabelling that should allow improvements in future antibiotic treatments of reported BLC allergy. Unlike all the studies related to penicillins reactivity, in which the chemical structures of their antigenic determinants are already established, the chemistry of other β-lactam antibiotics lacks this information.

Allergic reactions to these families may occur by sensitisation to them or to determinants that share similar chemical chains to those of penicillins. Different epitopes have not yet been identified. This study develops a multiplex sensitive immunoassay for the in vitro determination of both specific IgG and IgE levels by using major and minor determinants of a wide variety of less-studied β-lactam antibiotics, such as cefuroxime, cefotaxime, ceftriaxone, meropenem and aztreonam. It demonstrates that all the produced antigenic determinants are selective for detecting specific anti-β-lactam IgG in rabbit sera, but only minor determinants are able to detect specific anti-β-lactam IgE in human serum samples from allergic patients because the -lloyl determinants formed with this protocol are not recognised. This work also demonstrates that the carrier protein plays an important role in molecular recognition pattern terms as only the prepared H1-derived determinants were specifically recognised using allergic patient samples. Thus in patients with a history of a serious and potentially IgE-mediated reaction to a cephalosporin, it is critical to avoid re-exposure to the same cephalosporin, a cephalosporin compound that shares the same side chain, and even to other BLC sharing the same side chain, such as ceftazidime and cefadroxil and aztreonam.

These findings reveal that haptenisation of β-lactam antibiotics renders enhanced and interesting molecular recognition events, even when the β-lactam ring remains unmodified. This contribution may improve the current clinical diagnostics of allergies to antibiotics cephalosporin, carbapenem and monobactam together with drug provocation tests used as the gold standard. Indeed, future studies are needed to corroborate these in vitro findings with oral provocation tests.

## Supplementary information

Supplementary Information.
