# Supplementary material for: Neo-antigens for the serological diagnosis of IgE-mediated drug allergic reactions to antibiotics cephalosporin, carbapenem and monobactam
Source: Sci Rep. 2020 Sep 29;10:16037. doi: 10.1038/s41598-020-73109-w (PMC7525514; doi:10.1038/s41598-020-73109-w)
Supplement: Supplementary file 1 — Supplementary Information. [file 41598_2020_73109_MOESM1_ESM.docx]

**Neo-antigens for the serological diagnosis of IgE-mediated drug allergic reactions to antibiotics cephalosporin, carbapenem and monobactam**

Edurne Peña-Mendizabal^a^, Sergi Morais*^a,b,c^ and Ángel Maquieira*^a,b,c^

*^a^Instituto Interuniversitario de Investigación de Reconocimiento Molecular y Desarrollo Tecnológico, Universitat Politècnica de València-Universitat de València, Camino de vera s/n, 46022, Valencia, Spain.*

*^b^Departamento de Química, Universitat Politècnica de València, Camino de Vera s/n, 46022, Valencia, Spain*

*^c^Unidad Mixta UPV-La Fe, Nanomedicine and Sensors, IIS La Fe, Valencia, Spain*

**SUPPLEMENTARY INFORMATION**

[**EXPERIMENTAL** 4](#_Toc43996286)

[1. Instrumental methods 4](#_Toc43996287)

[2. Chemical and characterisation procedures 5](#_Toc43996288)

[Acidification of cephalosporin salts 5](#_Toc43996289)

[Acidification of ceftriaxone sodium salt hemi(heptahydrate) 5](#_Toc43996290)

[Acidification of cefuroxime sodium salt 6](#_Toc43996291)

[Acidification of cefotaxime sodium salt 6](#_Toc43996292)

[NMR spectra 8](#_Toc43996293)

[4-((E)-2-(((6R,7R)-2-carboxy-3-(((2-methyl-5,6-dioxo-1,2,5,6-tetrahydro-1,2,4-triazin-3-yl)thio)methyl)-8-oxo-5-thia-1-azabicyclo[4.2.0]oct-2-en-7-yl)amino)-1-(methoxyimino)-2-oxoethyl)thiazol-2-aminium 8](#_Toc43996294)

[(6R,7R)-3-((carbamoyloxy)methyl)-7-((E)-2-(furan-2-yl)-2-(methoxyimino)acetamido)-8-oxo-5-thia-1-azabicyclo[4.2.0]oct-2-ene-2-carboxylic acid monohydrate 9](#_Toc43996295)

[(6R,7R)-3-(acetoxymethyl)-7-((E)-2-(2-aminothiazol-4-yl)-2-(methoxyimino)acetamido)-8-oxo-5-thia-1-azabicyclo[4.2.0]oct-2-ene-2-carboxylic acid 10](#_Toc43996296)

[MS-MALDI-TOF spectra 11](#_Toc43996297)

[Control HSA 12](#_Toc43996298)

[Control H1 12](#_Toc43996299)

[Ceftriaxone determinants 13](#_Toc43996300)

[Meropenem determinants 14](#_Toc43996301)

[Aztreonam determinants 15](#_Toc43996302)

[Control samples 16](#_Toc43996303)

[Calibration curve for IgE 16](#_Toc43996304)

[Dot blot results of rabbit sera for the lloyl and llanyl determinants 18](#_Toc43996305)

[Affinity parameters of rabbit sera for the different antigenic determinants 19](#_Toc43996306)

[Dose-response affinity curves obtained with polyclonal rabbit IgG 21](#_Toc43996307)

# **EXPERIMENTAL**

## Instrumental methods

NMR Spectra: ^1^H and ^13^C-NMR spectra were recorded on a Bruker AVIIIHD NanoBay 400 MHz spectrometer using the TOPSPIN software. Proton and carbon chemical shifts (δH, δC) are quoted in ppm and referenced to tetramethylsilane with residual protonated solvent as the internal standard. Resonances are described using the following abbreviations; s (singlet), d (doublet), t (triplet), q (quartet), quin. (quintet), m (multiplet), br (broad), app (apparent), dd (doublet of doublets), etc. Coupling constants (J) are given in Hz and are rounded to the nearest 0.1 Hz.

Mass Spectra: high-resolution mass spectra were recorded by an AB SCIEX Triple TOF™ 5600 LC/MS/MS System. The employed LC system was an Agilent 1290 HPLC system. The analyses were performed using an Agilent EC-C_18_.The date acquisition was done in the positive mode within a mass range of 100–950 m/z. MS was done following an IDA acquisition method with the survey scan type (TOF-MS) and the dependent scan type (product ion) using 35 V of collision energy. Data were evaluated using qualitatively with the PeakView^TM^ software. The m/z values are reported in Daltons; the high-resolution values were calculated to four decimal places from the molecular formula, and all the found values were within a tolerance of 5 ppm.

Chromatography techniques: TLC was performed on Merck Glass TLC silica gel 60 F_254_ 0.2 mm precoated plates and visualised by ultraviolet light and potassium permanganate staining.

MS-MALDI-TOF: samples were analysed in a 5800 MALDI TOF-TOF (ABSciex) in the positive linear mode (1,500 shots every position) within a mass range of 15000-100000 m/z. For that purpose, 1.0 μL of every sample solution was spotted onto the MALDI plate. After the droplets were air-dried at room temperature, 1.0 μL of matrix (10 mg/mL of sinapinic acid in 0.1% TFA-ACN/H2O (7:3, v/v)) was added and allowed to air-dry at room temperature.

## Chemical and characterisation procedures

### Acidification of cephalosporin salts

#### Acidification of ceftriaxone sodium salt hemi(heptahydrate)

4-((E)-2-(((6R,7R)-2-carboxy-3-(((2-methyl-5,6-dioxo-1,2,5,6-tetrahydro-1,2,4-triazin-3-yl)thio)methyl)-8-oxo-5-thia-1-azabicyclo[4.2.0]oct-2-en-7-yl)amino)-1-(methoxyimino)-2-oxoethyl)thiazol-2-aminium

A dried round-bottomed flask (RBF) was filled with a solution of ceftriaxone sodium salt hemi(heptahydrate) (508.4 mg, 0.77 mmol, 1 eq) in H_2_O (7 mL). Then, the solution was acidified with 280 µL of HCl 6 M to pH 1 and vacuum filtration was performed. Finally, the product was dried in a high vacuum to give a yellow solid and a 99% yield. ^1^H NMR (400 MHz, DMSO-d_6_): δ 13.73 (s, 1H), 11.87 (s, 1H), 9.58 (d, J = 8.1 Hz, 1H), 7.23 (s, 2H), 6.73 (s, 1H), 5.76 (dd, J = 7.9, 4.8 Hz, 1H), 5.13 (d, J = 4.8 Hz, 1H), 4.38 (d, J = 13.3 Hz, 1H), 4.09 (d, J = 13.2 Hz, 1H), 3.83 (s, 3H), 3.73 (d, J = 18.4 Hz, 1H), 3.59 (s, 3H), 3.55 (d, J = 18.7 Hz, 1H). ^13^C NMR (100 MHz, DMSO-d_6_): δ167.63, 164.39, 162.98, 160.34, 156.19, 153.23, 148.52, 125.92, 108.93, 61.89, 58.64, 56.98, 42.79, 32.04, 26.21. HRMS (ESI-TOF) m/z calculated for C_18_H_18_N_8_O_7_S_3_ ([M+H^+^]): 555.0533, found: 555.0542.

#### Acidification of cefuroxime sodium salt

(6R,7R)-3-((carbamoyloxy)methyl)-7-((E)-2-(furan-2-yl)-2-(methoxyimino)acetamido)-8-oxo-5-thia-1-azabicyclo[4.2.0]oct-2-ene-2-carboxylic acid monohydrate

A dried RBF was filled with a solution of cefuroxime sodium salt (301.8 mg, 0.68 mmol, 1 eq) in H_2_O (3 mL). Then the solution was acidified with 200 µL of HCl 6 M to pH 1 and vacuum filtration was performed. Finally, the product was dried in a high vacuum to give a yellow solid and a 79% yield. ^1^H NMR (400 MHz, DMSO-d_6_): δ 13.62 (s, 1H), 9.77 (d, J = 7.9 Hz, 1H), 7.84 (d, J = 1.2 Hz, 1H), 6.69 (d, J = 3.4 Hz, 1H), 6.65 – 6.61 (m, 1H), 5.79 (dd, J = 7.6, 4.8 Hz, 1H), 5.19 (d, J = 4.8 Hz, 1H), 4.89 (d, J = 12.9 Hz, 1H), 4.61 (d, J = 12.8 Hz, 1H), 3.89 (s, 3H), 3.63 (d, J = 17.6 Hz, 2H), 3.45 (d, J = 17.6 Hz, 1H).^13^C NMR (100 MHz, DMSO-d_6_): δ 163.40, 162.84, 161.61, 156.43, 145.39, 144.91, 112.87, 112.02, 62.32, 62.27, 58.72, 57.37, 25.72. HRMS (ESI-TOF) m/z calculated for C_16_H_16_N_4_O_8_S ([M+H^+^]): 443.0867, found: 443.1064.

#### Acidification of cefotaxime sodium salt

(6R,7R)-3-(acetoxymethyl)-7-((E)-2-(2-aminothiazol-4-yl)-2-(methoxyimino)acetamido)-8-oxo-5-thia-1-azabicyclo[4.2.0]oct-2-ene-2-carboxylic acid

A dried RBF was filled with a solution of cefotaxime sodium salt (300.6 mg, 0.63 mmol, 1 eq) in H_2_O (4 mL). Then the solution was acidified with 160 µL of HCl 6 M to pH 1 and vacuum filtration was performed. Finally, the product was dried in a high vacuum to give a yellow solid and a 69 % yield. ^1^H NMR (400 MHz, DMSO-d_6_): δ 13.68 (s, 1H), 9.59 (d, J = 8.2 Hz, 1H), 7.21 (s, 2H), 6.74 (s, 1H), 5.79 (dd, J = 7.9, 4.9 Hz, 1H), 5.15 (d, J = 4.8 Hz, 1H), 4.99 (d, J = 12.8 Hz, 1H), 4.69 (d, J = 12.8 Hz, 1H), 3.84 (s, 3H), 3.62 (d, J = 18.1 Hz, 1H), 3.48 (d, J = 18.1 Hz, 1H), 2.03 (s, 3H). ^13^C NMR (100 MHz, DMSO-d_6_): δ 170.22, 168.46, 164.15, 162.89, 148.69, 126.40, 123.00, 108.95, 62.70, 61.93, 58.67, 57.48, 54.92, 25.77, 20.58. HRMS (ESI-TOF) m/z calculated for C_16_H_17_N_5_O_7_S_2_ ([M+H^+^]): 456.0642, found: 456.0646.

### NMR spectra

4-((E)-2-(((6R,7R)-2-carboxy-3-(((2-methyl-5,6-dioxo-1,2,5,6-tetrahydro-1,2,4-triazin-3-yl)thio)methyl)-8-oxo-5-thia-1-azabicyclo[4.2.0]oct-2-en-7-yl)amino)-1-(methoxyimino)-2-oxoethyl)thiazol-2-aminium

#### (6R,7R)-3-((carbamoyloxy)methyl)-7-((E)-2-(furan-2-yl)-2-(methoxyimino)acetamido)-8-oxo-5-thia-1-azabicyclo[4.2.0]oct-2-ene-2-carboxylic acid monohydrate

#### (6R,7R)-3-(acetoxymethyl)-7-((E)-2-(2-aminothiazol-4-yl)-2-(methoxyimino)acetamido)-8-oxo-5-thia-1-azabicyclo[4.2.0]oct-2-ene-2-carboxylic acid

### MS-MALDI-TOF spectra

The histone H1 used in this study was an isolated lysine rich fraction of mainly subfraction f1, in which other subfractions were still present as multiple peaks in the spectra. Although H1 was unable to be analysed by MS-MALDI-TOF, antigens HSA and H1 were prepared following the same experimental procedure, and both proteins presented approximately 60 free lysine residues. These are the reasons why the molar ratios (β-lactam/carrier protein) of antigens H1 were estimated to be the same as those obtained for the respective HSA antigens. The shown spectra corresponded to antigens HSA. Both the control HSA and H1 spectra were included.

The molecular weight (MW) of each antigenic determinant was calculated from the peak centroid of the peaks according to the following equation: [MW(determinant)-MW(protein)]/MW(hapten). The incremental change in molecular weight due to the incorporation of hapten molecules into protein corresponded to the number of hapten molecules per protein molecule.

#### Control HSA


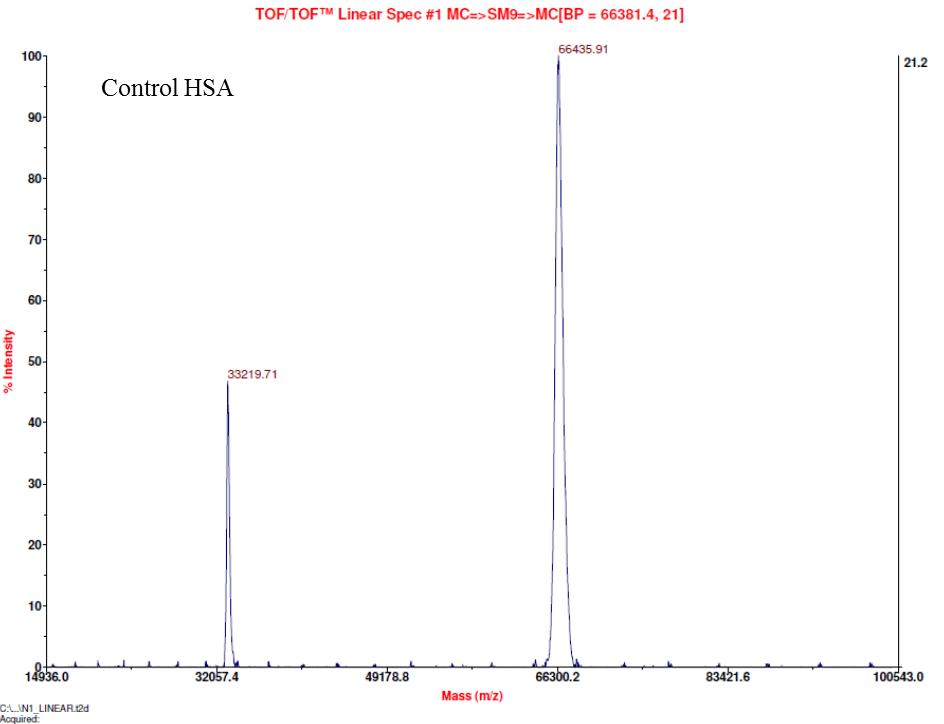


#### Control H1


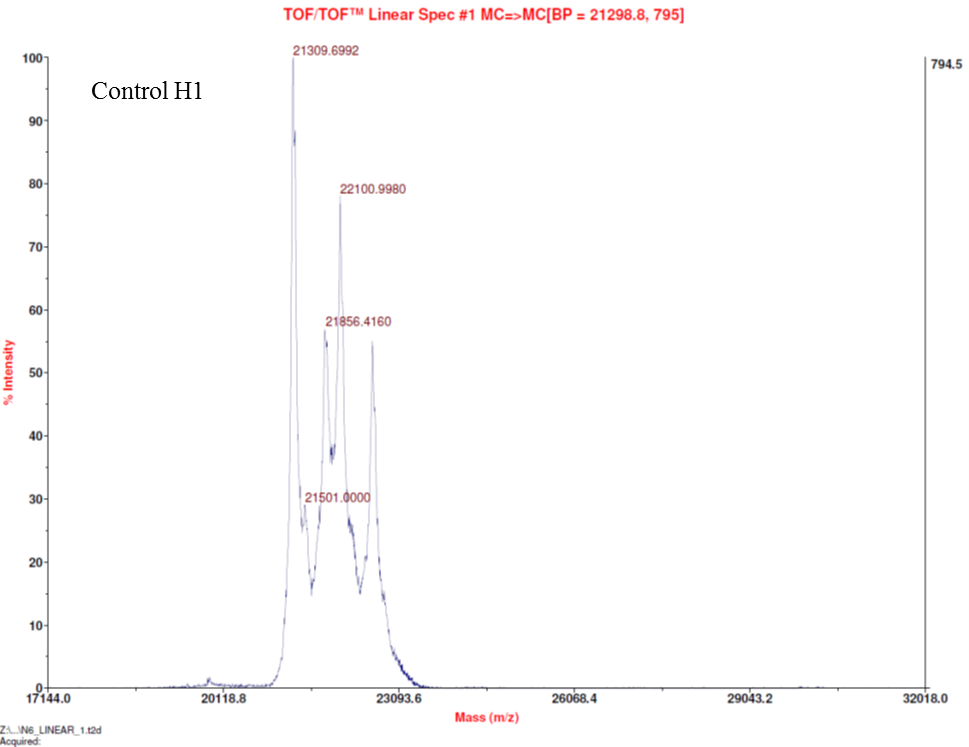


#### Ceftriaxone determinants


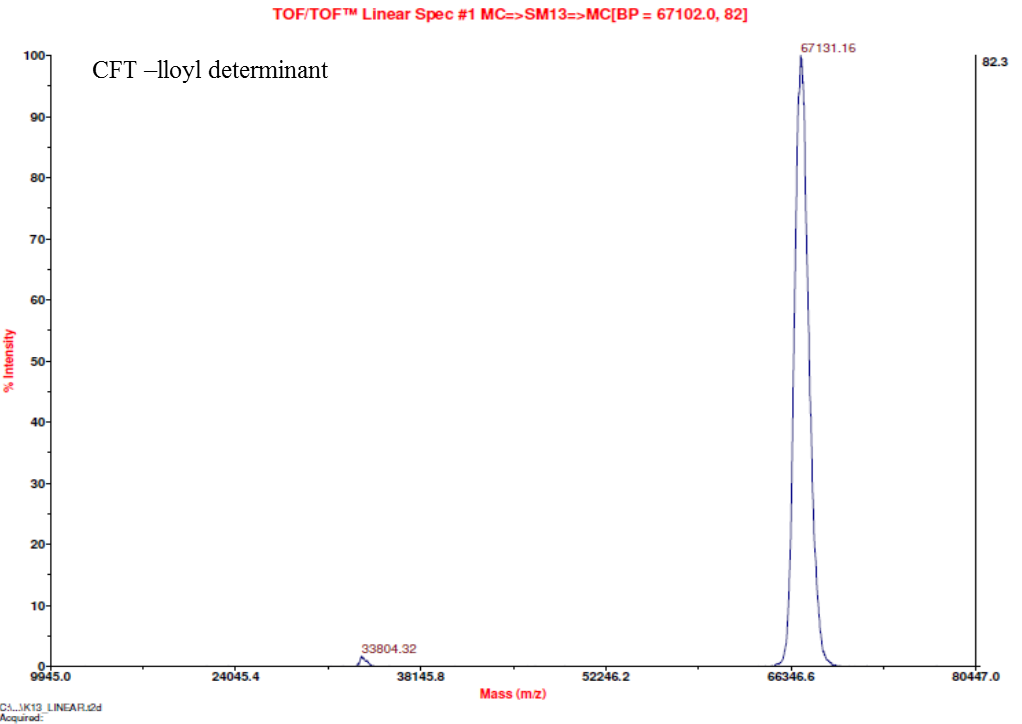


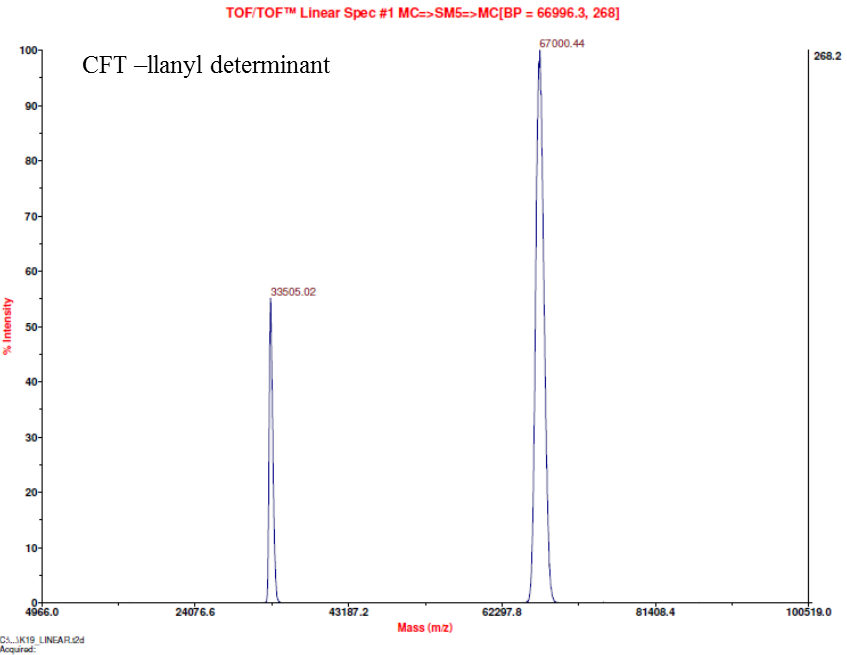


#### Meropenem determinants


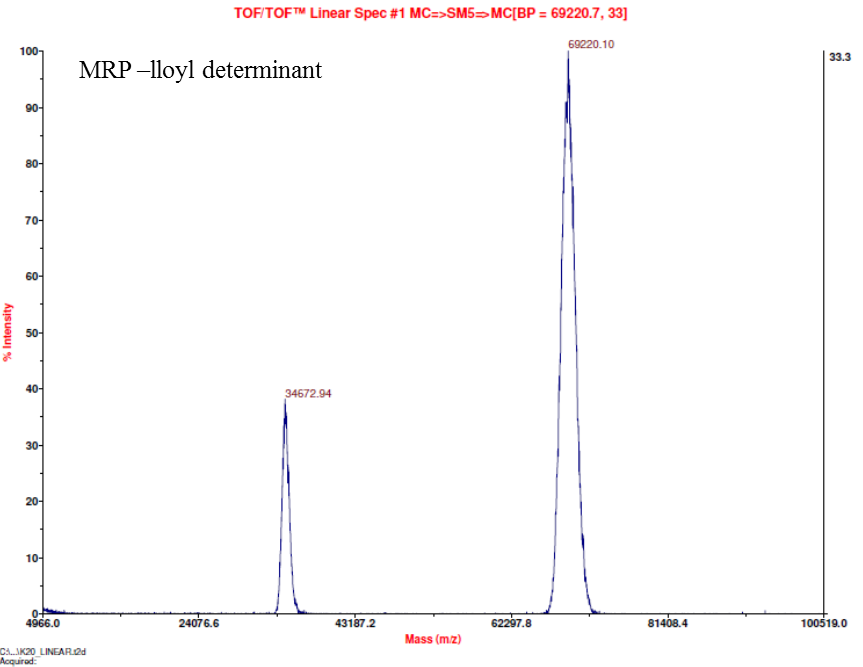


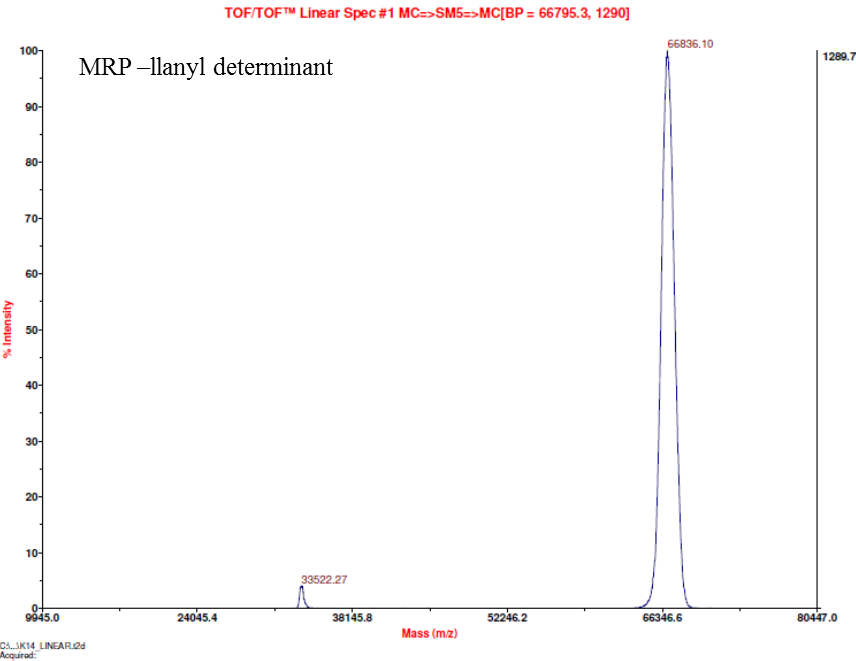


#### Aztreonam determinants


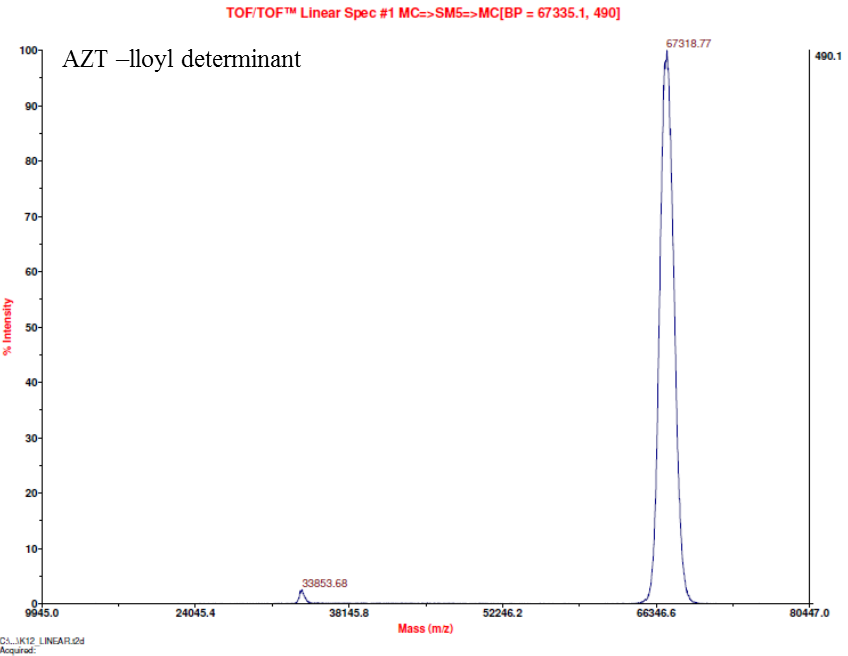


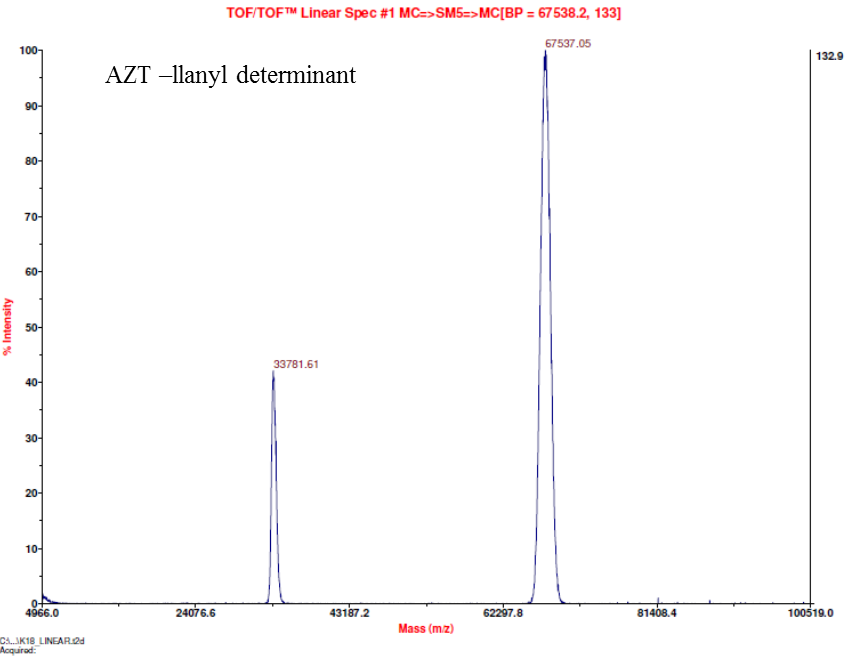


### Control samples

Supplementary Table S1: Clinical characteristics of the cohort of control patients.

| **Control**  **Number** | **Sex** | **Age**  **(Years)** |  | **Control**  **Number** | **Sex** | **Age**  **(Years)** |  | **Control**  **Number** | **Sex** | **Age**  **(Years)** |
| --- | --- | --- | --- | --- | --- | --- | --- | --- | --- | --- |
| 01 | M | 21 |  | 14 | M | 56 |  | 27 | M | 68 |
| 02 | F | 32 |  | 15 | M | 46 |  | 28 | F | 58 |
| 03 | M | 55 |  | 16 | F | 69 |  | 29 | F | 45 |
| 04 | F | 35 |  | 17 | F | 35 |  | 30 | F | 72 |
| 05 | F | 79 |  | 18 | F | 42 |  | 31 | F | 49 |
| 06 | F | 71 |  | 19 | M | 31 |  | 32 | F | 55 |
| 07 | F | 64 |  | 20 | F | 61 |  | 33 | F | 49 |
| 08 | F | 60 |  | 21 | F | 71 |  | 34 | F | 50 |
| 09 | M | 82 |  | 22 | M | 63 |  | 35 | F | 61 |
| 10 | F | 56 |  | 23 | F | 66 |  | 36 | F | 31 |
| 11 | M | 62 |  | 24 | F | 49 |  | 37 | F | 76 |
| 12 | M | 63 |  | 25 | F | 50 |  |  |  |  |
| 13 | M | 76 |  | 26 | F | 22 |  |  |  |  |

### Calibration curve for IgE

The calibration curve obtained for the IgE determination using the 3^rd^ WHO International Standard for serum IgE and the captured antibody Omalizumab is shown in Figure 4. The employed calibrators were 0, 0.35, 0.70, 3.50, 17.5 and 100 IU/mL. The standard data points (signal vs. semi-log concentration) were the mean of five curves performed in different days and on distinct disks. A four-parameter logistic (4PL) curve was fitted through points using the SigmaPlot 11 software.

The obtained data had a sensitivity (IC_50_) of 0.92±0.03 IU/mL, a slope of 1.24 and a regression coefficient (r^2^) of 0.9998. The limit of detection (LOD) was calculated as the concentration corresponding to the signal of the blank, plus 3-fold its relative standard deviation; 0.008 IU/mL. The limit of quantification (LOQ) was 0.06 IU/mL, with a relative standard deviation ranging from 3% to 15%. The concentration of the specific IgE for human serum samples was calculated with the calibration curve.

Supplementary Figure S1: Calibration curve for IgE.

Dot blot results of rabbit sera for the lloyl and llanyl determinants


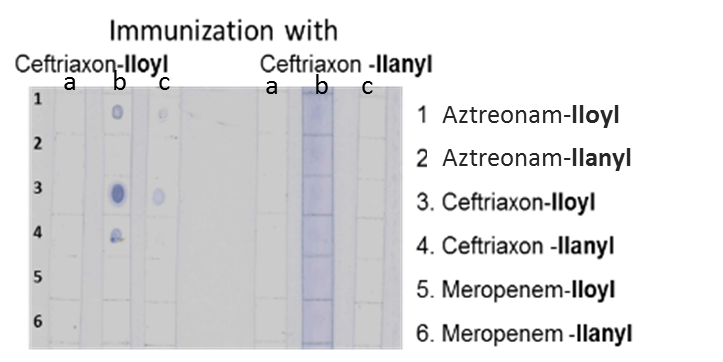


***
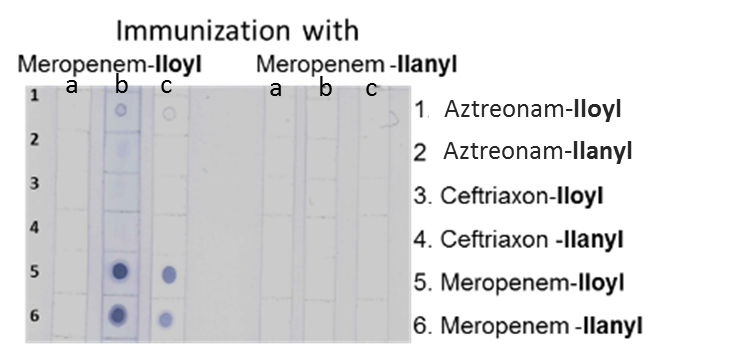
***

***
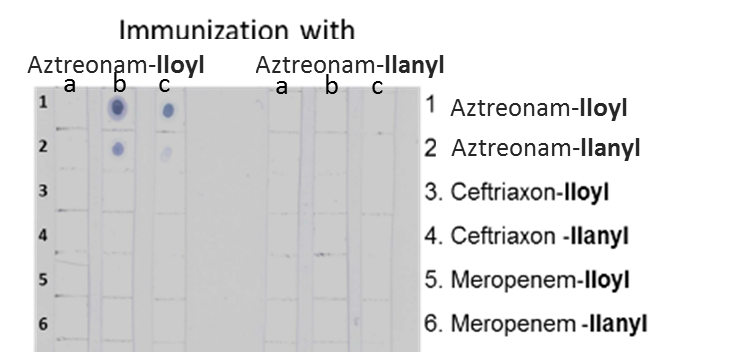
***

Supplementary Figures S2-S4: DotbBlot results using sera raised from immunised rabbit to CFT, MRP and AZT with the corresponding HSA antigens to CFT, MRP and AZT. a) pre-immune sera diluted at 1/100 (v/v), b) sera diluted at 1/100 (v/v) and c) 1/500 (v/v) in PBST.

### Affinity parameters of rabbit sera for the different antigenic determinants

Supplementary Table S2: The affinity parameters (K_d_^app^ and R^2^) values of the polyclonal rabbit antibodies raised towards the BLC families of the –lloyl determinants.

|  | -lloyl determinants | | | | | | | | |
| --- | --- | --- | --- | --- | --- | --- | --- | --- | --- |
|  | HSA | | | | | | | | |
|  | α-IgG-CFT | | | α-IgG-MRP | | | α-IgG-AZT | | |
|  | K_d_^app^ | Dilution factor | R^2^ | K_d_^app^ | Dilution factor | R^2^ | K_d_^app^ | Dilution factor | R^2^ |
| CFR | 9.896·10^-4^ | 1/1,011 | 0.9944 | 1.653·10^-3^ | 1/605 | 0.9951 | 5.083·10^-3^ | 1/197 | 0.9991 |
| CFT | 1.265·10^-3^ | 1/791 | 0.9989 | 4.978·10^-3^ | 1/201 | 0.9825 | NR | NR | NR |
| CF0 | 1.201·10^-3^ | 1/833 | 0.9927 | 1.898·10^-3^ | 1/527 | 0.9926 | 6.044·10^-3^ | 1/165 | 0.9989 |
| MRP | 4.515·10^-2^ | 1/22 | 0.9989 | 7.819·10^-3^ | 1/128 | 0.9994 | NR | NR | NR |
| AZT | 9.678·10^-4^ | 1/1,033 | 0.9854 | NR^a^ | NR | NR | 1.790·10^-4^ | 1/5,587 | 0.9963 |
|  | H1 | | | | | | | | |
|  | α-IgG-CFT | | | α-IgG-MRP | | | α-IgG-AZT | | |
|  | K_d_^app^ | Dilution factor | R^2^ | K_d_^app^ | Dilution factor | R^2^ | K_d_^app^ | Dilution factor | R^2^ |
| CFR | 7.304·10^-4^ | 1/1,369 | 0.9865 | 1.151·10^-3^ | 1/869 | 0.9982 | 3.985·10^-3^ | 1/251 | 0.9964 |
| CFT | 8.642·10^-4^ | 1/1,157 | 0.9939 | 1.362·10^-3^ | 1/734 | 0.9966 | 1.166·10^-3^ | 1/858 | 0.9605 |
| CF0 | 4.404·10^-4^ | 1/2,271 | 0.9975 | 1.871·10^-3^ | 1/534 | 0.9931 | 3.163·10^-3^ | 1/316 | 0.9971 |
| MRP | 4.515·10^-2^ | 1/22 | 0.9989 | NR | NR | NR | 3.258·10^-3^ | 1/307 | 0.9610 |
| AZT | NR | NR | NR | NR | NR | NR | 2.650·10^-4^ | 1/3,774 | 0.9893 |

K_d_^app^= Apparent affinity constant (or apparent binding affinity) determined as the concentration of the specific α-IgG sera to achieve 50% of the maximum signal (expressed as 1/dilution factor); R^2^= coefficient of determination, R-squared; NR^a^=Not recognised.

Supplementary Table S3: The affinity parameters (K_d_^app^ and R^2^) values of the polyclonal rabbit antibodies raised towards the BLC families of the –llanyl determinants.

|  | -llanyl determinants | | | | | | | | |
| --- | --- | --- | --- | --- | --- | --- | --- | --- | --- |
|  | HSA | | | | | | | | |
|  | α-IgG-CFT | | | α-IgG-MRP | | | α-IgG-AZT | | |
|  | K_d_^app^ | Dilution factor | R^2^ | K_d_^app^ | Dilution factor | R^2^ | K_d_^app^ | Dilution factor | R^2^ |
| CFR | 3.028·10^-4^ | 1/3,303 | 0.8971 | 2.619·10^-4^ | 1/3,818 | 0.6965 | 4.883·10^-4^4 | 1/2,048 | 0.6864 |
| CFT | 2.314·10^-3^ | 1/432 | 0.9966 | 2.352·10^-4^ | 1/4,252 | 0.6111 | NR | NR | NR |
| CF0 | NR^a^ | NR | NR | 2.770·10^-2^ | 1/36 | 0.9884 | NR | NR | NR |
| MRP | 9.891·10^-3^ | 1/101 | 0.9475 | 1.339·10^-3^ | 1/747 | 0.9953 | NR | NR | NR |
| AZT | NR | NR | NR | NR | NR | NR | 4.488·10^-4^ | 1/2,228 | 0.9984 |
|  | H1 | | | | | | | | |
|  | α-IgG-CFT | | | α-IgG-MRP | | | α-IgG-AZT | | |
|  | K_d_^app^ | Dilution factor | R^2^ | K_d_^app^ | Dilution factor | R^2^ | K_d_^app^ | Dilution factor | R^2^ |
| CFR | 1.245·10^-3^ | 1/803 | 0.9933 | NR | NR | NR | 2.230·10^-4^ | 1/4,484 | 0.9689 |
| CFT | 4.612·10^-4^ | 1/2,168 | 0.9910 | 7.823·10^-4^ | 1/1,278 | 0.9930 | 1.191·10^-4^ | 1/8,396 | 0.9940 |
| CF0 | 5.009·10^-4^ | 1/1,996 | 0.9765 | 6.620·10^-4^ | 1/1,511 | 0.9799 | 9.131·10^-4^ | 1/1,095 | 0.9918 |
| MRP | 2.963·10^-4^ | 1/3,375 | 0.9460 | 6.958·10^-4^ | 1/1,437 | 0.9917 | 1.090·10^-3^ | 1/917 | 0.9620 |
| AZT | 8.663·10^-4^ | 1/1,154 | 0.9724 | 1.508·10^-3^ | 1/663 | 0.9522 | 4.712·10^-4^ | 1/2,122 | 0.9659 |

K_d_^app^= Apparent affinity constant (or apparent binding affinity) determined as the concentration of the specific α-IgG sera to achieve 50% of the maximum signal (expressed as 1/dilution factor); R^2^= coefficient of determination, R-squared; NR^a^=Not recognised.

### Dose-response affinity curves obtained with polyclonal rabbit IgG


Supplementary Figure S5: Dose-response curves obtained for the –lloyl determinants with polyclonal rabbit IgG. Dilution factors: 1/250, 1/1,000, 1/4,000 and 1/16,000 and blank, PBS-T. a) HSA determinants, b) H1 determinants.


Supplementary Figure S6: Dose-response curves obtained for the –llanyl determinants with polyclonal rabbit IgG. Dilution factors: 1/250, 1/1,000, 1/4,000 and 1/16,000 and blank, PBS-T. a) HSA determinants, b) H1 determinants
